# Prevalence of Transfusion-Transmitted Viral Infections Among Blood Donors in Riyadh, Saudi Arabia: A Comparative Analysis of Nucleic Acid Testing and Serological Screening

**DOI:** 10.3390/jcm15166131

**Published:** 2026-08-07

**Authors:** Rana M. Aldhalaan, Mohammed Raihan Sajid, Layla Raddaoui, Rimah Abdullah Saleem, Nour Odeh, Rawan Elshaer, Salman Aldosari, Muhammad Faisal Ikram

**Affiliations:** 1 College of Medicine, Alfaisal University, Riyadh 11533, Saudi Arabia; 2 Blood Transfusion Services Center, Health Supported Services Center, Ministry of Health, Riyadh 12382, Saudi Arabia; 3 Department of Pathology, College of Medicine, Alfaisal University, Riyadh 11533, Saudi Arabia; 4 Department of Biochemistry and Molecular Medicine, College of Medicine, Alfaisal University, Riyadh 11533, Saudi Arabia; 5 Department of Anatomy, College of Medicine, Alfaisal University, Riyadh 11533, Saudi Arabia

**Keywords:** blood donors, transfusion-transmitted infections, nucleic acid testing, serology, hepatitis B virus, hepatitis C virus, HIV, Saudi Arabia

## Abstract

**Background/Objectives**: Transfusion-transmitted infections (TTIs) remain an important concern in blood safety. Nucleic acid amplification testing (NAT) shortens the diagnostic window period relative to conventional serological assays. This study aimed to determine the prevalence of Hepatitis B virus(HBV), Hepatitis C virus (HCV), and Human immunodeficiency virus (HIV) among blood donors and to evaluate concordance between NAT and conventional serological screening methods. **Methods**: A retrospective cross-sectional study was conducted at Riyadh Regional Laboratory from May 2021 to May 2022 and included 48,901 blood donors. Donor samples were tested for HBV, HCV, and HIV using serological assays (chemiluminescent immunoassays on the Abbott Alinity s system) and NAT. Data were analyzed using SPSS. **Results**: The prevalence of HCV was 0.149% by serological testing and 0.061% by NAT. HBV prevalence was 0.252% by serology and 0.264% by NAT, while HIV prevalence was 0.041% and 0.037%, respectively. NAT identified 25 seronegative but NAT-reactive donations, including 13 HBV, 11 HCV, and 1 HIV donation. A statistically significant difference between NAT and serology was observed for HCV (*p* < 0.001, McNemar’s test), but not for HBV (*p* = 0.180) or HIV (*p* = 0.317). **Conclusions**: The prevalence of major viral TTIs among blood donors was low, with HBV being the most frequently detected infectious agent. NAT and serological assays provide complementary information and support the continued use of combined screening strategies to enhance transfusion safety.

## 1. Introduction

Blood transfusion is an essential component of modern medical practice, particularly for managing acute blood loss, surgical procedures, and severe anemia. Despite its life-saving benefits, transfusion carries a risk of transfusion-transmitted infections (TTIs), which remain a major patient safety concern. Hepatitis B virus (HBV), hepatitis C virus (HCV), and human immunodeficiency virus (HIV) are among the most clinically significant TTIs, all capable of causing chronic disease and substantial morbidity [1].

To minimize these risks, blood transfusion services have implemented strict donor selection criteria and comprehensive screening strategies. The World Health Organization recommends mandatory screening of all donated blood for markers of major transfusion-transmitted pathogens, including HBV, HCV, HIV, and *Treponema pallidum* [2]. Serological testing, based on the detection of viral antigens and antibodies, has significantly reduced the transmission of these infections. However, serological testing remains limited by the diagnostic window period, during which recently infected individuals may test negative despite being infectious [3,4].

Despite major advances in transfusion medicine and blood donor screening, HBV, HCV, and HIV remain important public health concerns worldwide because of their potential transmission through blood products [2]. Transmission of these viruses through contaminated blood products can result in chronic liver disease, cirrhosis, hepatocellular carcinoma, acquired immunodeficiency syndrome, and increased mortality [5,6,7]. Consequently, ensuring the microbiological safety of donated blood remains a fundamental objective of blood transfusion services globally [2].

HBV remains one of the most prevalent blood-borne viral infections worldwide, although universal vaccination programs have substantially reduced its incidence in many countries. HCV remains a major cause of chronic liver disease globally and is often characterized by a prolonged asymptomatic phase, leading to delayed diagnosis and continued transmission. HIV prevalence among blood donors is generally low in many countries because of strict donor selection procedures; however, because of the severe clinical consequences associated with infection, even a small residual risk remains a significant concern for transfusion services.

Serological testing has historically served as the cornerstone of blood donor screening programs. Screening assays based on the detection of viral antigens and antibodies have significantly reduced the transmission of HBV, HCV, and HIV through blood transfusion. Nevertheless, serological testing has inherent limitations because detection depends on the development of measurable antigen or antibody levels following infection. During the early stages of infection, individuals may harbor and transmit the virus despite testing negative by conventional serological methods. This interval, commonly termed as the diagnostic or serological window period, remains one of the major challenges in blood safety. Although the exact duration varies according to the assay used, NAT substantially shortens this window period for HIV, HBV, and HCV, thereby reducing the residual risk of transfusion-transmitted infection [4,8].

Nucleic acid testing (NAT) has emerged as a highly sensitive molecular method because it detects viral genetic material directly, allowing for the earlier detection of infection and reducing the diagnostic window period. The addition of NAT to routine screening improves detection and reduces the residual risk of TTIs [4,8,9,10]. Nevertheless, implementation is associated with higher costs, technical complexity, and the need for specialized infrastructure, leading to variability in adoption across healthcare systems. In many settings, NAT is used alongside serological testing to maximize blood safety [4,8,9].

Globally, studies have demonstrated considerable variation in the prevalence of TTIs among blood donors. A retrospective study in Ethiopia reported seroprevalence rates of 3.8% for HIV, 4.7% for HBV, and 0.7% for HCV. In contrast, lower prevalence rates were reported in China, with 0.08% for HIV, 0.51% for HBV, and 0.20% for HCV. Studies from Kyrgyzstan and other regions have also shown differing prevalence patterns, reflecting geographic and population differences. These variations highlight the importance of region-specific data to guide screening strategies and improve blood safety [11,12,13].

Within Saudi Arabia, previous studies have also reported regional variation in HBV, HCV, and HIV prevalence among blood donors. In addition, previous studies have demonstrated that NAT improves the detection of HBV and reduces residual risk compared with serological testing alone, supporting its incorporation into routine blood donor screening. More recent regional and national data have reported relatively low prevalence rates of HBV, HCV, and HIV among blood donors, although differences persist between regions [14,15,16].

Given these regional variations and the ongoing need to optimize blood safety, the continuous evaluation of donor screening strategies remains essential. Although previous studies have evaluated transfusion-transmitted infections among blood donors in Saudi Arabia, most were conducted in individual regions or focused primarily on serological screening. Data directly comparing NAT with conventional serological assays in large contemporary donor populations remain limited. Therefore, the present study aimed to determine the frequency of HBV, HCV, and HIV among healthy blood donors at Riyadh Regional Laboratory (RRL) and to evaluate concordance between NAT and conventional serological screening methods for detecting these infections.

## 2. Materials and Methods

### 2.1. Study Setting and Design

This retrospective cross-sectional study was conducted at the Riyadh Regional Laboratory (RRL), a central blood bank facility supplying screened blood and blood products to transfusion services across Riyadh Province. The study period extended over 12 months, from May 2021 to May 2022.

### 2.2. Ethical Approval

Ethical approval was obtained from the Institutional Review Board of RRL (Reference No. H1RI-17-Nov21-03) on 13 November 2022. The study was retrospective in nature and involved the analysis of de-identified data from routine blood donor screening. The IRB approved the waiver of additional informed consent for this retrospective analysis, as all data were de-identified and no additional interventions were performed beyond routine screening. The study was conducted in accordance with the Declaration of Helsinki.

### 2.3. Study Population and Data Collection

A total of 48,901 unique voluntary blood donors were included in this analysis. Donors who donated multiple times during the study period were counted only once, and their first donation during the study period was included in the analysis to avoid duplication. This approach ensures that the unit of analysis is the individual donor rather than the donation event. Donations were collected at the central blood bank or during mobile blood donation campaigns. All eligible donor samples routinely screened during the study period were included in the analysis.

### 2.4. Laboratory Testing

Whole blood was collected according to standard blood bank operating procedures under aseptic conditions. Serum samples were used for serological testing, while plasma samples were used for nucleic acid testing (NAT).

Serological screening was performed using the Abbott Alinity s system (Abbott Laboratories, Abbott Park, IL, USA), which employs chemiluminescent immunoassay (CMIA) technology for the detection of the following markers:

- HBV: Hepatitis B surface antigen (HBsAg) using the Alinity i HBsAg assay (Abbott Laboratories, Abbott Park, IL, USA); additionally, samples were tested for anti-HBc (Alinity i Anti-HBc II assay, Abbott Laboratories, Abbott Park, IL, USA).
- HCV: Anti-HCV antibodies using the Alinity i Anti-HCV assay (Abbott Laboratories, Abbott Park, IL, USA).
- HIV: HIV-1 p24 antigen and anti-HIV-1/HIV-2 antibodies using the Alinity i HIV Ag/Ab Combo assay (Abbott Laboratories, Abbott Park, IL, USA).

NAT was conducted using the Procleix Panther System (Grifols, Los Angeles, CA, USA) with the Procleix Ultrio Elite assay, based on transcription-mediated amplification for the detection of HIV RNA, HCV RNA, and HBV DNA. Additional testing was performed using the cobas 6800 system (Roche Diagnostics, Basel, Switzerland) with the cobas MPX assay, which detects HIV-1, HIV-2, HCV RNA, and HBV DNA using real-time PCR.

Screening Algorithm:

All donor samples underwent parallel testing using both serological assays and NAT. The screening algorithm was as follows:

Initial screening: All samples were tested simultaneously using serological assays (Abbott Alinity s system) and NAT (Procleix Panther System with Ultrio Elite assay).

Additional NAT: To maximize sensitivity and allow cross-platform verification, all samples were also tested on the cobas 6800 system with the cobas MPX assay as a secondary NAT platform.

Reactive results: Any sample that was reactive on either serology or NAT was considered initially reactive and underwent repeat testing in duplicate using the same method.

Discordant results: Samples with discordant results (serology-positive/NAT-negative or serology-negative/NAT-positive) were retested on both platforms.

Final classification: A sample was considered positive if it was repeatedly reactive on at least one testing platform. Confirmatory testing (e.g., Western blot, line immunoassay) and donor follow-up were not performed as part of routine screening and were not available for this retrospective analysis.

### 2.5. Data Management and Statistical Analysis

Serological and NAT results were recorded and analyzed to identify seronegative but NAT-reactive samples (NAT yield). Quality assurance was maintained in accordance with national standards.

Data were analyzed using SPSS version 20 (SPSS Inc., Chicago, IL, USA). Continuous variables were expressed as mean ± standard deviation, while categorical variables were presented as frequencies and percentages. Comparisons between serological testing and NAT results were made using McNemar’s test for paired binary data, which is the appropriate statistical test for comparing two diagnostic methods on the same samples. Cohen’s kappa coefficient was calculated to measure the level of agreement between the two testing methods. Statistical significance was set at *p* < 0.05. Samples with invalid results (e.g., equipment error, insufficient sample volume) were excluded from the analysis. In total, 23 samples (0.047%) had invalid results and were excluded, leaving 48,901 valid donor records for analysis. No indeterminate results were reported for any of the assays used, as the testing platforms provide clear positive/negative discrimination. Missing data were not an issue for the laboratory results, as all included samples had complete serology and NAT results.

## 3. Results

Data from 48,901 blood donors who donated blood at RRL from May 2021 to May 2022 were collected and analyzed. The mean age of donors was 32.31 ± 7.8 years (range, 18–60 years). The majority of donors were male (46,853; 95.8%), with females comprising only 2048 (4.2%) of the cohort. Repeat donors accounted for 29,559 (60.4%) of donations, while first-time donors numbered 19,342 (39.6%). Of the donors, 23,081 (47.2%) were Saudi and 25,820 (52.8%) were non-Saudi (Table 1).

Overall prevalence of transfusion-transmitted infections was low (Table 2). HBV demonstrated the highest prevalence among donors, followed by HCV, while HIV showed the lowest prevalence. NAT detected a slightly higher number of HBV cases compared with serology, whereas lower NAT detection rates were observed for HCV and HIV.

Agreement between NAT and serological screening results is summarized in Table 3. Concordance was highest for HBV and HIV, while greater discordance was observed for HCV. Among HCV-serology-positive donors, 19 were NAT-positive and 54 were NAT-negative. In addition, 11 donors were NAT-positive but serology-negative for HCV.

Positive, negative, and overall agreement were calculated using serological testing as the comparator. NAT identified 25 seronegative but NAT-reactive donations, including 13 HBV, 11 HCV, and 1 HIV donation. These values reflect agreement between screening methods and should not be interpreted as the analytical sensitivity or specificity of NAT.

Table 4 presents the prevalence of HCV, HBV, and HIV stratified by donor nationality, showing the number and percentage of positive donors identified by serology and NAT among Saudi and non-Saudi donors separately. When stratified by nationality, non-Saudi donors showed a higher prevalence of HCV than Saudi donors by both serology (0.198% vs. 0.095%; *p* = 0.003, chi-square test) and NAT (0.077% vs. 0.043%; *p* = 0.012). HBV prevalence was similar between Saudi and non-Saudi donors (serology: 0.273% vs. 0.232%, *p* = 0.37; NAT: 0.286% vs. 0.244%, *p* = 0.35), while HIV prevalence remained low in both groups (serology: 0.048% vs. 0.035%, *p* = 0.48; NAT: 0.052% vs. 0.023%, *p* = 0.09).

## 4. Discussion

In this retrospective study of 48,901 blood donors at Riyadh Regional Laboratory, the overall prevalence of HBV, HCV, and HIV was low by both serological testing and NAT. HBV was the most frequently detected infectious agent, whereas HIV was rare. A slightly higher prevalence of HCV was observed among non-Saudi donors compared with Saudi donors, which may reflect differences in background prevalence, country of origin, healthcare access, socioeconomic factors, and prior exposure risk. Overall, these findings are consistent with recent Saudi blood donor studies showing a relatively low burden of major transfusion-transmitted viral infections [15,16].

Compared with international blood donor studies, the prevalence in the present cohort appears low. Prior studies from Ethiopia, China, and Kyrgyzstan have reported variable rates of HBV, HCV, HIV, and syphilis among blood donors, reflecting differences in background endemicity, donor recruitment practices, public health infrastructure, and screening policies. These comparisons should be interpreted cautiously because donor populations differ substantially in age, sex distribution, donation type, repeat donation status, and local eligibility criteria. Nevertheless, the consistently low prevalence in the present cohort supports the effectiveness of the current RRL screening system and highlights the value of ongoing regional surveillance over reliance on international estimates [11,12,13].

The interpretation of these prevalence estimates should also account for the characteristics of the donor population. Voluntary blood donors generally represent a healthier, more selected population than the general public because individuals with known infection, recent high-risk exposures, acute illness, or other deferral criteria are excluded before donation. In this study, voluntary donors were non-remunerated, and the cohort included both first-time (39.6%) and repeat (60.4%) donors. All donations were from voluntary, non-remunerated individuals as per Saudi blood banking regulations. As a result, donor-based prevalence estimates are useful for blood safety surveillance but should not be interpreted as direct estimates of community prevalence. This distinction is important when comparing the present findings with population-based studies or with data from countries that use different donor recruitment models [2,3].

The low prevalence of HCV observed in this donor cohort likely reflects the combined effects of donor selection, mandatory screening, infection-control measures, and public health awareness regarding viral hepatitis transmission. Blood donors represent a pre-screened and relatively low-risk population because they undergo pre-donation assessment and risk-based screening before donation. Therefore, HCV prevalence among blood donors is expected to be lower than that observed in the general population or in higher-risk groups. In addition, improvements in healthcare-associated infection control, safer injection practices, and routine screening of donated blood have likely contributed to the reduction in transfusion-related HCV risk over time. Continued surveillance remains important, particularly because HCV infection may remain asymptomatic for long periods and can only be detected reliably through systematic screening [2,15,16].

The slightly higher HCV prevalence among non-Saudi donors also warrants attention. Saudi Arabia has a large expatriate population, and blood donors may originate from countries with varying background prevalence of viral hepatitis. Differences in childhood vaccination programs, prior healthcare exposure, access to screening, and public health infrastructure in countries of origin may influence the observed prevalence of HCV among non-Saudi donors. However, this finding should be interpreted cautiously because the study did not include detailed data on country of origin, duration of residence in Saudi Arabia, prior HCV treatment, or behavioral risk factors. Future studies incorporating more detailed donor-level demographic and epidemiologic data would be useful to better understand the factors contributing to nationality-based differences in HCV prevalence.

Another important consideration is that prevalence estimates based on serological testing and NAT are not directly interchangeable. Serological assays may identify markers of prior exposure, immune response, or established infection, whereas NAT detects viral nucleic acid and therefore more directly reflects current viremia. This distinction is especially relevant for HCV, where antibody reactivity may persist even after spontaneous clearance or treatment, while HCV RNA may no longer be detectable. For this reason, lower NAT positivity compared with serology does not necessarily indicate under-detection by NAT; rather, it may indicate that a proportion of serology-reactive donors did not have active circulating virus at the time of donation [4,8].

HBV was the most frequently detected viral infection in this cohort, although the overall prevalence remained low. This pattern is consistent with recent Saudi blood donor data showing that HBV-related markers account for a large proportion of TTI-positive donations. The relatively low HBV prevalence in the present study may reflect the long-term impact of national HBV vaccination programs, improved donor selection, and routine screening protocols. Universal HBV vaccination has likely contributed to a sustained reduction in HBV transmission among younger generations, while donor screening has further reduced the risk of transfusion-transmitted HBV. Nevertheless, HBV remains particularly important in blood donor screening because of the possibility of occult HBV infection, in which HBV DNA may be detectable despite absent or low-level serological markers [17].

The predominance of HBV among the detected infections is also biologically and epidemiologically plausible. HBV is efficiently transmitted through blood and body fluids and can persist chronically, making it an important target for donor screening even in populations with declining prevalence. Although vaccination has reduced the burden of HBV in younger age groups, older donors or donors from regions with incomplete vaccination coverage may still carry infection or serological markers of prior exposure. In addition, occult HBV infection remains a recognized challenge for transfusion services because HBV DNA may be present at low levels even when conventional serological markers are negative or equivocal [17,18]. These features help explain why HBV continues to receive particular attention in blood safety programs despite overall reductions in prevalence [4,5,8].

The slightly higher NAT positivity for HBV compared with serology may therefore be clinically relevant. NAT can detect HBV DNA during early infection before serological markers become detectable, and it may also identify occult HBV infection in donors who are negative by conventional serological screening [4,8,17]. This supports the role of NAT as an important adjunctive test in HBV screening, particularly in settings where maintaining a high level of blood safety is essential. However, because confirmatory testing and donor follow-up were not available in this retrospective analysis, the clinical significance of discordant HBV results cannot be fully determined. These findings should therefore be interpreted as screening results rather than definitive diagnostic classification [4,8].

HIV prevalence was very low among both Saudi and non-Saudi donors. This finding is reassuring from a transfusion safety perspective, as transfusion of contaminated blood remains an efficient route of HIV transmission despite the low prevalence in screened donor populations. The low number of HIV-positive donors is consistent with the generally low burden of HIV reported in Saudi Arabia [19] and likely reflects the effectiveness of donor selection, risk-based deferral, serological testing, and molecular screening. Nevertheless, because even a single infected donation can have major clinical consequences, maintaining robust screening strategies remains essential. In low-prevalence settings, the main value of combined screening is not necessarily detecting a large number of positive cases, but rather minimizing the residual risk of rare but clinically serious contaminations entering the blood supply [2,4,8].

Comparison of NAT and serological screening revealed discordant results across the three infections. Agreement was highest for HBV and HIV, whereas greater discordance was observed for HCV. For HCV, a substantial number of serology-reactive samples were NAT-negative. This finding should not be interpreted as indicating poor analytical sensitivity of NAT. Rather, it likely reflects the different biological targets detected by each method. Serological assays detect antigen or antibody responses and may remain reactive after resolved infection or prior exposure, whereas NAT detects circulating viral nucleic acid and is more closely associated with active viremia. Therefore, a donor with previous HCV exposure or resolved infection may remain serology-positive while being NAT-negative. Conversely, NAT-positive/serology-negative results may represent early window-period infection, low-level viremia, or cases in which serological markers are not yet detectable [4,8].

Discordance between NAT and serological testing reinforces the complementary role of these methods in blood donor screening. Serological assays remain essential because they can identify donors with evidence of prior exposure or established infection, while NAT provides an additional layer of protection by detecting viral nucleic acid earlier in the course of infection. The combined use of both approaches can therefore reduce the risk of window-period transmission and improve the overall safety of the blood supply [20]. This is particularly relevant in large-volume blood centers, where even a very low residual risk may translate into clinically meaningful consequences given the large number of donations processed [2,4,8].

From a public health perspective, the continued use of NAT alongside serological screening supports national efforts to reduce the residual risk of transfusion-transmitted infections. Although NAT requires specialized infrastructure, trained personnel, and higher costs, its ability to detect viral nucleic acid during the diagnostic window period provides an important safety advantage. The findings of this study support the continued integration of molecular and serological screening approaches within Saudi blood transfusion services. At the same time, future evaluations should consider cost-effectiveness, testing algorithms, confirmatory strategies, and donor notification pathways, particularly for discordant results. Strengthening these components may help optimize donor management, improve surveillance, and maintain public confidence in the safety of the blood supply [2,4,8].

Operationally, NAT implementation in routine blood donor screening requires careful balancing of safety benefits, cost, laboratory capacity, and workflow [4,10]. NAT platforms require specialized equipment, trained personnel, quality assurance procedures, and reliable turnaround times to avoid delays in releasing blood products. However, these operational demands must be weighed against the potential consequences of transfusion-transmitted infection, particularly in high-volume centers serving large populations. The findings of this study suggest that NAT contributes useful complementary information, especially for identifying potentially contaminated donations that may be missed during the serological window period. Future policy decisions should therefore consider not only the number of additional infections detected, but also recipient safety, donor notification, public confidence, and the broader role of NAT in national surveillance systems [2,4,8].

Future multicenter studies are needed to better characterize the national epidemiology of HBV, HCV, and HIV among blood donors in Saudi Arabia. Studies that include confirmatory testing, donor follow-up, repeat donor status, vaccination history, country of origin, and behavioral risk factors would provide a more complete understanding of infection patterns and discordant screening results. Such data would also help us to determine the true NAT yield and clarify whether NAT-positive/serology-negative cases represent window-period infections, occult infection, or other forms of low-level viremia. These additional data would be valuable for refining screening policies and ensuring that blood safety strategies remain evidence-based and responsive to changing epidemiologic trends.

A standardized approach to discordant screening results would strengthen the clinical and public health value of future studies. Donors with NAT-positive/serology-negative or serology-positive/NAT-negative results may require confirmatory testing, repeat sampling, counseling, and clear documentation of donor eligibility for future donations. Linking laboratory results with donor history and follow-up testing would allow blood centers to distinguish between false reactivity, resolved infection, occult infection, and true window-period infection. Such information would improve estimation of true NAT yield and help define the most appropriate screening algorithms for Saudi blood donation services [2,4,8].

### 4.1. Strengths

This study has several strengths. First, the large sample size (48,901 donors) provides robust estimates of prevalence and agreement between screening methods. Second, the use of two NAT platforms (Procleix Panther and cobas 6800) enhances the reliability of molecular testing results. Third, the inclusion of nationality-stratified data allows for subgroup comparisons. Fourth, the study provides contemporary data (2021–2022) from a major regional blood center in Saudi Arabia, contributing to the limited literature on NAT versus serology comparison in the region.

### 4.2. Limitations

This study has several limitations. First, it was conducted at a single center, which may limit the generalizability of the findings to other regions of Saudi Arabia. Second, blood donors represent a selected population and may not reflect the prevalence of these infections in the general population. Third, the retrospective design limited the availability of clinical, demographic, and behavioral risk-factor data, including prior infection, treatment history, vaccination status, and repeat donor status. Fourth, confirmatory testing and donor follow-up data were not available; therefore, discordant NAT and serological results could not be further characterized. The 25 seronegative but NAT-reactive samples are reported as screening results rather than confirmed NAT-yield cases. Without confirmatory testing, it is not possible to definitively classify these as true window-period infections, occult infections, or false-positive NAT results. Finally, because NAT and serological assays detect different biological targets, comparisons between the two methods should be interpreted as screening agreement rather than definitive diagnostic performance.

## 5. Conclusions

This study demonstrated low prevalence of HBV, HCV, and HIV among 48,901 blood donors at Riyadh Regional Laboratory, with prevalence rates of 0.264% for HBV, 0.061% for HCV, and 0.037% for HIV by NAT. HBV was the most frequently detected agent, while HIV remained rare. NAT identified 25 seronegative but NAT-reactive donations (13 HBV, 11 HCV, and 1 HIV), representing potential window-period or occult infections that would have been missed by serology alone. These findings support the continued use of combined serological and NAT-based screening to enhance blood safety and reduce the residual risk of transfusion-transmitted infections. We recommend that blood centers in Saudi Arabia maintain dual screening strategies and establish standardized protocols for confirmatory testing and donor follow-up of discordant results. Future multicenter studies with comprehensive donor-level demographic data, confirmatory testing, and long-term donor follow-up are needed to better characterize discordant screening results and inform national blood safety policy.

## Figures and Tables

**Table 1:** Characteristics of blood donors (N = 48,901).

Variable	Value
Total donors	48,901
Mean age, years	32.31 ± 7.8
Age range, years	18–60
Sex, n (%)	
Male	46,853 (95.8%)
Female	2048 (4.2%)
Donor type, n (%)	
First-time donors	19,342 (39.6%)
Repeat donors	29,559 (60.4%)
Nationality, n (%)	
Saudi donors	23,081 (47.2%)
Non-Saudi donors	25,820 (52.8%)

**Table 2:** Prevalence of HCV, HBV, and HIV by serological testing and NAT.

Infection	Serology Positive, n (%)	NAT Positive, n (%)	*p*-Value (McNemar’s Test)
HCV	73 (0.149%)	30 (0.061%)	<0.001
HBV	123 (0.252%)	129 (0.264%)	0.18
HIV	20 (0.041%)	18 (0.037%)	0.317

**Table 3:** Agreement between NAT and serological screening results.

Infection	Serology+/NAT+	Serology+/NAT−	Serology−/NAT+	Serology−/NAT−	Positive Agreement (%)	Negative Agreement (%)	Overall Agreement (%)	Kappa (95% CI)
HCV	19	54	11	48,817	26	99.98	99.87	0.38 (0.26–0.50)
HBV	116	7	13	48,765	94.3	99.97	99.96	0.92 (0.87–0.97)
HIV	17	3	1	48,880	85	100	99.99	0.89 (0.77–1.00)

**Table 4:** Prevalence of HCV, HBV, and HIV by nationality and testing method.

Infection	Saudi Serology, n (%)	Saudi NAT, n (%)	Non-Saudi Serology, n (%)	Non-Saudi NAT, n (%)
HCV	22 (0.095%)	10 (0.043%)	51 (0.198%)	20 (0.077%)
HBV	63 (0.273%)	66 (0.286%)	60 (0.232%)	63 (0.244%)
HIV	11 (0.048%)	12 (0.052%)	9 (0.035%)	6 (0.023%)

Statistical analysis demonstrated a significant difference between NAT and serological testing for HCV (*p* < 0.001, McNemar’s test). However, no statistically significant differences were observed for HBV (*p* = 0.180) or HIV (*p* = 0.317).

## Data Availability

The data supporting the findings of this study are available from the corresponding author upon reasonable request, subject to institutional data-sharing policies.
